# Topological, Mechanical and Biological Properties of Ti6Al4V Scaffolds for Bone Tissue Regeneration Fabricated with Reused Powders via Electron Beam Melting

**DOI:** 10.3390/ma14010224

**Published:** 2021-01-05

**Authors:** Maria Laura Gatto, Riccardo Groppo, Nora Bloise, Lorenzo Fassina, Livia Visai, Manuela Galati, Luca Iuliano, Paolo Mengucci

**Affiliations:** 1 Department SIMAU, Università Politecnica delle Marche, Via Brecce Bianche 12, 60131 Ancona, Italy; p.mengucci@univpm.it; 2 Department of Engineering “Enzo Ferrari”, Università di Modena e Reggio Emilia, 41121 Modena, Italy; riccardo.groppo@unimore.it; 3 Department of Molecular Medicine, Centre for Health Technologies (CHT), INSTM UdR of Pavia, University of Pavia, 27100 Pavia, Italy; nora.bloise@unipv.it (N.B.); livia.visai@unipv.it (L.V.); 4 Department of Occupational Medicine, Toxicology and Environmental Risks, ICS Maugeri, IRCCS, 27100 Pavia, Italy; 5 Department of Electrical, Computer and Biomedical Engineering, Centre for Health Technologies (CHT), University of Pavia, Via Ferrata 5, 27100 Pavia, Italy; lorenzo.fassina@unipv.it; 6 Department of Management and Production Engineering (DIGEP), Politecnico di Torino, 10129 Torino, Italy; manuela.galati@polito.it (M.G.); luca.iuliano@polito.it (L.I.)

**Keywords:** titanium implants, additive manufacturing, reused powder, unit cell topology, tissue engineering, mechanical properties, stem cells, surface functionalization

## Abstract

Cellularized scaffold is emerging as the preferred solution for tissue regeneration and restoration of damaged functionalities. However, the high cost of preclinical studies creates a gap between investigation and the device market for the biomedical industry. In this work, bone-tailored scaffolds based on the Ti6Al4V alloy manufactured by electron beam melting (EBM) technology with reused powder were investigated, aiming to overcome issues connected to the high cost of preclinical studies. Two different elementary unit cell scaffold geometries, namely diamond (DO) and rhombic dodecahedron (RD), were adopted, while surface functionalization was performed by coating scaffolds with single layers of polycaprolactone (PCL) or with mixture of polycaprolactone and 20 wt.% hydroxyapatite (PCL/HA). The mechanical and biological performances of the produced scaffolds were investigated, and the results were compared to software simulation and experimental evidence available in literature. Good mechanical properties and a favorable environment for cell growth were obtained for all combinations of scaffold geometry and surface functionalization. In conclusion, powder recycling provides a viable practice for the biomedical industry to strongly reduce preclinical costs without altering biomechanical performance.

## 1. Introduction

Recently, an approach to bone tissue regeneration based on cellularized-scaffold implantation was used to overcome the risks connected to bone grafts when self-healing fails [1].

The focal point of scaffolds for bone tissue regeneration is unit cell topology which influences mechanical and biological performance [1]. Suitable mechanical properties are required in load-bearing applications while suitable mass transport properties are necessary for biological activities [2]. Both these features relate to porosity, pore size and pore interconnectivity, that in turn are determined by scaffold elementary unit cell geometry. Therefore, scaffold design must be balanced to allow nutrients and oxygen supply, metabolic waste removal, cell penetration and reduction of stress shielding to prevent mechanical failure [3,4].

Two main elementary unit cell geometries for the scaffold are proposed in the literature: (a) diagonal symmetrical and (b) midline symmetrical. Finite element analysis (FEA) [5] showed that a porous structure with midline symmetry exhibited superior compressive performances than diagonal symmetry, while the opposite trend was observed in the case of torsion. Therefore, rhombic dodecahedron (RD) unit cell geometry (midline symmetrical) is well suited for load-bearing implants, while diamond (DO) unit cell geometry (diagonal symmetrical) is preferable for implants subjected to torsional forces. In addition, numerical analysis by computational fluid dynamics (CFD) [6] has demonstrated that DO shows advantages in implant fixation (greater tortuosity and larger aperture), cell growth environment (more appropriate adhesion areas), and tissue regeneration (due to regular mechanical stimulation), while RD shows a superior mass transport performance (higher flow velocity value). Therefore, DO and RD elementary unit cells are valid alternatives for bone tissue regeneration.

Accurate control of the anatomical shape, size and topology of the scaffold’s elementary unit cell can be efficiently obtained by additive manufacturing (AM) technology alternatives to traditional subtractive methods. Typically, AM processes for metal alloys consist of melting powders through an energy source (laser or electron beam) in a layer-by-layer process, according to a CAD file [7,8]. Laser-manufactured parts show lower surface roughness in comparison to electron beam melting (EBM) products, that generally exhibit residual stresses and gas contamination. Laser-melting technology is widely used for dental implants [9,10], while EBM is commonly employed in a patient’s customized bone implants [11,12].

Titanium alloys have an elastic modulus in the range 50–118 GPa, closer to natural cortical bone (10–30 GPa) than any other widely investigated alloy for permanent implant, such as cobalt chromium (240 GPa) and stainless steel (216 GPa) [13]. However, the difference in elastic modulus between cortical bone and titanium alloys leads to stress shielding with reabsorption of the unstressed bone portion and consequent risk of implant failure [14].

A combination of EBM technology and Ti6Al4V alloy has been investigated by Popov et al. [11], that produced biomedical patient-specific implants for clavicle, mandibular bone, foot osteotomy and hip. In the reported clinical cases, EBM revealed a reduction of time in operation and patient recovery.

Despite the key advantages of combining EBM technology with Ti-based alloys, only a small percentage of tissue engineering research in this field achieves clinical application, due to the high cost of preclinical studies. To overcome this gap, Martinez−Marquez et al. [15] proposed a step model to ensure the cost-effectiveness of customized scaffolds. In particular, they proposed material feedstock recycling as the first step in cost reduction. Material feedstock in EBM processes can affect up to 31% of the cost of built parts [16]. Therefore, recycling Ti6Al4V metal powder represents an affordable solution to reduce waste and save costs [17]. However, powder reuse means repeated preheating steps and prolonged thermal treatments in a vacuum during the EBM process that can induce changes in the reused powder, in terms of chemical composition, morphology and physical properties [18]. In Ti6Al4V alloy thermal cycling due to powder reuse induces variations in particle size distribution and oxygen uptake that can lead it to exceed the O < 0.2 wt.% condition required by ASTM F2924-14 [19]. Oxygen stabilizes the α-Ti phase which pins dislocation during part deformation. Thus, with powder reuse, ultimate yield strength slightly increases, while ductility decreases [18,20,21].

In addition to elementary unit cell geometry and scaffold production technology, the engineering aspect to be improved for implant success is surface functionalization, which must fulfill scaffold–bone interface requirements. The most common functionalization treatment is coating the scaffold’s surface with a layer of biocompatible material or a combination of biocompatible compounds [22,23,24]. Surface coating plays a double role: (a) avoids the release of toxic ions [3,4,25,26] and (b) improves implant osteointegration.

Currently, the researchers’ interest is focused on synthetic bioresorbable and biocompatible polymers such as polylactide (PLA), polycaprolactone (PCL) and polyglycolide (PGA) [27]. Among them, PCL is the most promising due to slow degradation time (>24 months) with respect to PLA (12–16 months) and PGA (6–12 months) [28]. However, to overcome the PCL drawbacks concerning structural (elevated hydrophobicity) and biological aspects (poor bioactivity and low cell adhesion), PCL is usually combined with ceramic materials such as hydroxyapatite (HA). HA shows excellent biocompatibility, bioactivity and osteoconductive properties, allowing enhanced bone formation and implant-site recovery [29,30].

In this paper, the elementary unit cell geometry, surface functionalization, mechanical properties and biological response of scaffolds based on Ti6Al4V alloy, produced with a mixture of reused and virgin new powders by the EBM technology, were considered for bone tissue regeneration. The aim of the work is to fabricate suitable scaffolds for in vivo perspectives starting from industrial EBM process based on cost and waste reduction in order to overcome issues connected to the high costs of preclinical studies. Starting from reused powder blended with virgin new powder as the raw material of the EBM process, scaffolds with DO and RD elementary unit cell geometry were produced. After production, scaffolds were submitted to topological and mechanical characterization to compare their performances with the experimental and simulated results reported in literature [5] for identical structures (same alloy composition, production technology and elementary unit cell geometries). Afterwards, scaffolds were coated with a single layer of PCL or PCL/HA for surface functionalization and then submitted to biological tests. Human mesenchymal stem cell cultures for 24 h and 4 days were used as a fast check of the scaffold’s biological response. The role of unremoved residual powder in the scaffold core on mechanical and biological behavior was also considered. Results evidenced mechanical and biological performance of scaffolds produced by EBM industrial methods fully comparable to the literature data from scaffolds produced by virgin new powder, thus providing an indication for viable industrial processes for cost reduction of preclinical studies.

## 2. Materials and Methods

### 2.1. Material

Scaffolds were produced using Arcam Ti6Al4V powder. The raw material was a mixture of Arcam powder in recycled and virgin new conditions blended in unknown proportions. In this paper, from now on, the blended raw powder is indicated as P1, while the virgin new powder, used as reference, is indicated as P2. As provided by the manufacturer (Arcam website [31]), the virgin new powder P2 has a particle size in the range 45–106 µm, density 2.47 g/cm^3^ and the nominal chemical composition reported in Table 1.

### 2.2. Scaffold Geometry

**Figure 1:**
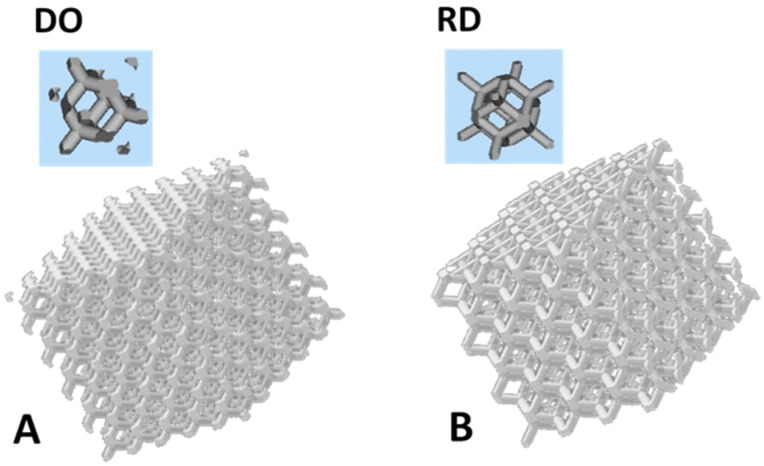
Schematics of the scaffolds’ elementary unit cell geometry: (**A**) diamond (DO) and (**B**) rhombic dodecahedron (RD).

The scaffolds’ local geometries adopted in this study are schematically shown in Figure 1. Scaffolds were designed using Magics 21.0 in diamond (DO, Figure 1A) and rhombic dodecahedron (RD, Figure 1B) elementary unit cell. Scaffold volume, independently on elementary unit cell geometry, corresponds to a cube 1 cm side length.

Nominal porosity and strut size are reported in Table 2 for diagonal symmetrical (DO) and midline symmetrical (RD) elementary unit cells. Theoretical porosity is constant (80%) for both geometries, while strut size changes on the basis of pore size and interconnectivity.

### 2.3. Scaffold Manufacturing

Scaffolds were additively manufactured using Arcam A2X electron beam melting (EBM) system. The entire procedure was carried out in a vacuum with the powder bed heated to 750 °C before the production process to induce partial sintering of powder particles. Sintering improves powder thermal conductivity and mechanical strength, thus allowing production of parts with a reduced number of supports or even without any supporting structure [32]. Processing parameters are reported in Table 3. After production and cooling, scaffolds were cleaned by Ti6Al4V ELI powder blasting at 4 bar pressure.

### 2.4. Scaffold Porosity and Residual Powder Volume

After powder blasting, some unremoved residual powder was still present in the inner central part of the scaffold. Residual powder was visible by visual inspection. Observations carried out by optical (Nikon OPTIPHOT-100, Tokyo, Japan) and scanning electron microscopy confirmed the presence of residual powder that partially and sometimes totally occluded pores in the scaffold core. The unremoved residual powder was partially sintered during scaffold production and could not be removed even after repeated blasting processes.

Scaffold porosity resulting from the production process was experimentally obtained by X-ray computed tomography (XCT) (Bruker Skyscan 1174 system, Kontich, Belgium) measurements performed on a slice cut from a scaffold external part, free of residual powder. The external slice total volume was 160 mm^3^.

The total volume of residual powder was estimated from experimental measurement of scaffold mass by using precision balance and five weighing operations. The mass of residual powder (m_pw_) was obtained from Equation (1):

(1) $$m_{s}\;={\;m}_{\mathrm{Ti}}\;+\;m_{\mathrm{pw}}$$

where m_s_ is scaffold mass, m_Ti_ mass of struts calculated from porosity data and alloy nominal density (ρ_Ti_ = 4.43 g/cm^3^) and m_pw_ total mass of unremoved residual powder. Residual powder volume (V_pw_) was calculated from residual powder density ρ_pw_ = 0.68 ρ_Ti_ = 3.012 g/cm^3^, as suggested by Tolochko et al. [33].

In order to check distribution of residual powder and presence of porosity occlusions in the scaffold core, scaffolds were cut by diamond blade at half height (about 0.5 cm below the upper scaffold surface). After acetone cleaning in ultrasonic system followed by air jetting, the scaffold was observed by scanning electron microscope.

### 2.5. Surface Functionalization

Surface functionalization was performed by coating the scaffold with a single layer of: (a) polycaprolactone (PCL) or (b) a mixture of polycaprolactone and 20% hydroxyapatite (PCL/HA). The coating operation was carried out by manually dipping the scaffold in the bath solution for 20 s, followed by air drying. The solvent used in the bath solution was tetrahydrofuran (THF, CH_2_Cl_2_), selected after C. Bordes et al. [34]. Chemical grade powders of polycaprolactone (PCL, Eurocoating S.p.a.) and hydroxyapatite (HA, Boc Sciences, Inc., Shirley, NY, USA) were used as solutes. The bath solution was obtained by gradually melting PCL and PCL/HA powders in THF solvent under magnetic stirring at room temperature.

### 2.6. Morphological, Structural and Mechanical Characterization

Morphological characterization of blended powder (P1) and reference virgin powder (P2) as well as of the DO and RD scaffolds was carried out by Zeiss Supra 40 field emission scanning electron microscope (FESEM) (Carl Zeiss AG, Oberkochen, Germany) equipped with Bruker Z200 energy dispersive microanalysis (EDS) (Billerica, MA, USA) and Tescan Vega 3 scanning electron microscope (SEM) (Brno, Czech Republic) equipped with EDAX Elements EDS system. Starting from FESEM images, powder mean grain size was evaluated by ImageJ software (version 1.52a, NIH, Bethesda, MD, USA [35]).

Scaffolds in DO and RD geometries were morphologically characterized by 3D light microscopy (3D LM) and FESEM observations.

Scaffold porosity was experimentally obtained by XCT analysis carried out in Bruker Skyscan 1174 system at V = 40 kV, I = 800 µA with Cu-Kα radiation source, pixel size 13.8 μm, rotation step 0.2°, total rotation angle 360°, exposure time per projection 8 s, aluminum filter (1 mm) for optimization of X-ray energy transmission. Projections were processed by SkyScan reconstruction program NRecon, collecting stacks of cross-sectional slices generated with the following settings: misalignment compensation = −3, smoothing = 3, ring artefacts reduction = 5, beam hardening correction = 8%. The 3D reconstructions of DO and RD slices were carried out by VG Studio MAX 1.2 software (Volume Graphics, Heidelberg, Germany).

Structural information from powders and scaffolds were obtained by X-ray diffraction (XRD), while EDS microanalysis was used to estimate chemical composition.

A Bruker D8 Advance diffractometer operating at V = 40 kV and I = 40 mA, with Cu-Kα radiation, in the angular range 2θ = 34°–43° was used for XRD measurements. Pattern analysis was carried out by DIFFRAC.EVA software package including ICDD—PDF 2 license for search/match analysis. Shape analysis of XRD peaks was carried out by OriginPro (version 8.5) software package.

EDS analysis was carried out by a Bruker Z200 microanalysis system installed in a Zeiss Supra 40 FESEM. Chemical composition by EDS were obtained by averaging data taken from three different sample areas observed at same magnification (1000×). Results are reported as average value (AV) and standard deviation (SD) calculated from the three measurements.

Topographic maps were obtained by Nikon LV 150 Confovis Microscope (Tokyo, Japan). Scaffold roughness parameters were measured from topographic maps, according to ISO 25178-603 [36].

Mechanical performance of uncoated scaffold was investigated by uniaxial compressive tests carried out in Instron 5567 system (5 kN load cell, 0.5 mm/min speed) (Norwood, MA, USA) in accordance with the standard ISO 13314:2011 [37]. For each scaffold geometry, four cubic samples 10 × 10 × 10 mm^3^ were tested. The results of compressive tests are plotted as a stress–strain curve from which were calculated: (a) maximum stress value at first peak, assumed as compression strength, after Li et al. [5], (b) Young modulus and (c) compressive strength at 40% strain. Mechanical parameters estimated from stress–strain curves allowed the comparison of results obtained in this work to literature data.

### 2.7. Biological Characterization

#### 2.7.1. Cell Culture Conditions

Human mesenchymal stem cells derived from bone marrow were isolated and phenotypically analyzed to assess their mesenchymal properties according to the International Society for Cellular Therapy by a method previously described [38,39]. The study was conducted in accordance with the Institution Review Board of Fondazione IRCCS Policlinico San Matteo and the University of Pavia (2011). Written informed consent was obtained from all participants enrolled in the study. Cells used in all experiments were mainly at passage 4–5.

Cells were grown at 37 °C in 5% CO_2_ humidified atmosphere, maintained in low-glucose DMEM (Dulbecco’s modified Eagle’s) supplemented with 10% FBS, 1% glutamine, 1% penicillin-streptomycin and amphotericin B (Lonza Group Ltd., Basel, Switzerland).

Before cell seeding, the scaffold was sterilized in an autoclave (Vapor Matic 770) at 120 °C for 20 min, washed with phosphate-buffered saline (PBS 1X), while coated scaffolds were sterilized by immersing in 70% ethanol bath for 20 min. Subsequent scaffolds were placed inside a standard 24-well-plate (Corning Inc., Corning, NY, USA), washed several times with sterile distillated water followed by PBS 1X and finally dried under ultraviolet light exposure for 40 min in order to prevent contamination. A cell suspension of 5 × 10^4^ was placed on the top of each scaffold. After 0.5 h of incubation, 1.3 mL of culture medium was added to the scaffold covering. The hMSC seeded and cultured on tissue culture plates (TCPS) wells were inserted as control.

#### 2.7.2. Cell Viability Assay

Cell viability on different scaffolds was estimated by using resazurin-based assay (TOX8-1KT Sigma Aldrich, St. Louis, MO, USA) after 1 and 4 days of incubation, as previously reported [40]. According to manufacturer instructions, resazurin solution (Sigma-Aldrich) was added in 1:10 ratio with respect to culture volume to each well plate, and incubated for 3 h at 37 °C in 5% CO_2_. At the end of incubation time, absorbance was measured at 570 and 600 nm wavelengths using a microplate reader (Bio-Rad Laboratories, Hercules, CA, USA). Each biological experiment was performed in triplicate and in at least three separate tests. Results were expressed as mean value ± standard deviation. Statistical analysis of hMSC viability data at 24 h and 4 days was performed using the GraphPad Prism (version 5.00 for Windows, GraphPad Software, San Diego, CA, USA) software package. Two-way ANOVA analysis was carried out to check the influence of geometry and surface treatments on cell viability at 24 h and 4 days, separately. Since two-way ANOVA analysis did not provide any significant results, t-tests were performed for DO and RD scaffold geometries on the following parameters: uncoated, PCL coated and PCL/HA coated scaffolds and vice versa, for both the cell viability times. Statistical significance was analyzed at: *p* < 0.05 *.

#### 2.7.3. Focal Adhesion Kinase—Enzyme-Linked Immunosorbent Assay

The activation of the Focal Adhesion Kinase (FAK) was measured by human phospho-FAK enzyme-linked immunosorbent assay (ELISA) kit (Human Phospho-FAK (Y397) and Total FAK ELISA, RayBiotech, Inc., Peachtree Corners, GA, USA). After 24 h from seeding, cell-seeded samples were washed extensively with sterile PBS to remove culture medium and lysed with ice-cold lysis buffer (50 mM Tris pH 7.5, 50 mM NaCl, 5 mM ethylenediaminetetraacetic acid, 0.1% Triton, and 1 mM sodium orthovanadate) for 30 min on ice. Afterwards, lysates were centrifuged at 13,000 rpm for 15 min at 4 °C and supernatants were analyzed by ELISA kit in accordance with manufacturer’s instructions. Data were expressed as mean value with standard deviation. Statistical analysis was performed using two-way ANOVA, to understand if geometry and surface treatments have statistically significative effects on the phospho-FAK on the total FAK signal. The software package used was GraphPad Prism (version 5.00 for Windows, GraphPad Software, San Diego, CA, USA) and differences were considered significant for *p* < 0.05 *. A graph was obtained normalizing the phospho-FAK on the total FAK signal.

#### 2.7.4. FESEM Observation

After 24 h and 4 days of incubation, cell seeded onto scaffolds were treated as previously described [41]. The specimens were fixed with 2.5% (*v*/*v*) glutaraldehyde solution in 0.1 M Na-cacodylate buffer (pH = 7.2) for 1 h at 4 °C, washed with Na-cacodylate buffer and then dehydrated at room temperature in ethanol gradient series up to 100%. Samples were frozen for at least one day and then lyophilized 6 h to complete dehydration. Finally, scaffolds were sputter-coated with gold and observed by FESEM.

## 3. Results

### 3.1. Structural and Morphological Characterization of Powders

**Figure 2:**
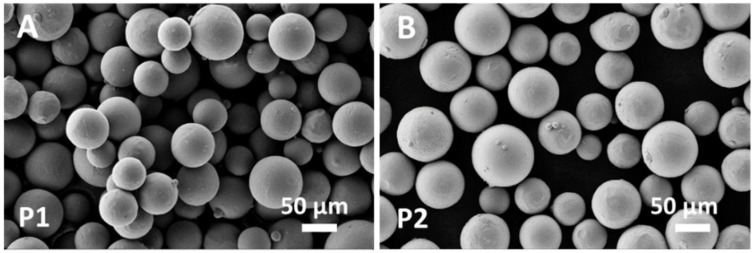
FESEM images of powders: (**A**) blended P1 and (**B**) virgin new P2.

FESEM observation allowed the morphology of the raw blended powder P1 to be compared to the reference virgin new powder P2 (Figure 2). Spherically shaped particles were visible in both P1 (Figure 2A) and P2 (Figure 2B). However, particle aggregation was clearly visible in P1 (Figure 2A), due to joint neck formation at the particle contact points, caused by the heating and cooling cycle for powder reuse.

The virgin new powder P2 (Figure 2B) is formed of spherical particles with small satellites and a size in the range of 40–110 µm in agreement with the values provided by the manufacturer. The blended powder P1 (Figure 2A) was also formed of spherical particles with a few residual satellites and a size in the range of 50–130 µm. Aggregation of spherical particles with neck formation was evident in P1 (Figure 2A).

The experimental chemical composition obtained by EDS analysis of P1 and P2 powders is reported in Table 4. Within experimental uncertainties, both powders had similar average chemical composition.

**Figure 3:**
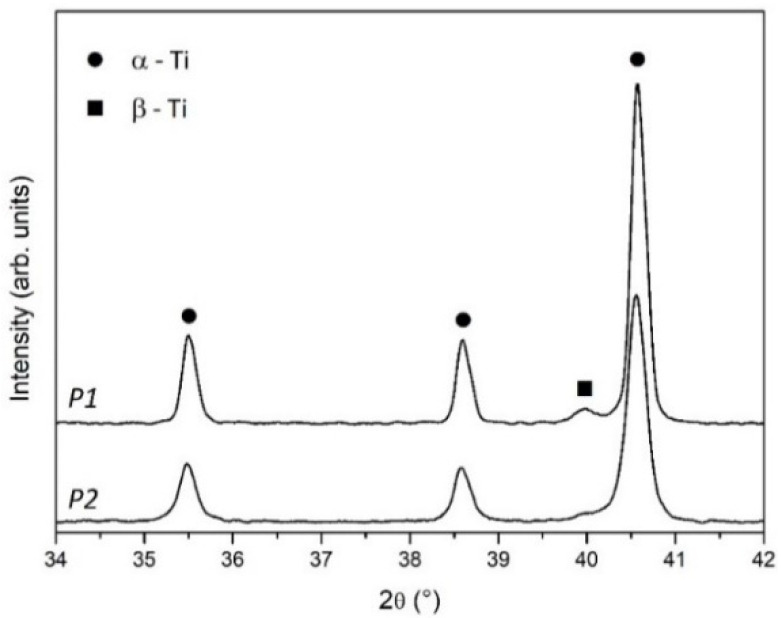
XRD patterns of P1 and P2 powders. Full dot—α-Ti, full square—β-Ti.

XRD patterns of powders are reported in Figure 3. Peak position of α-Ti (ICDD file n. 44-1294) and β-Ti (ICDD file n. 44-1288) phases are indicated by full dots and full squares, respectively.

Powders are mainly formed of α-Ti with a small contribution of β-Ti, which is more evident in P1, due to the recycled part of the blended powder.

### 3.2. Scaffold Characterization

#### 3.2.1. Geometry

**Figure 4:**
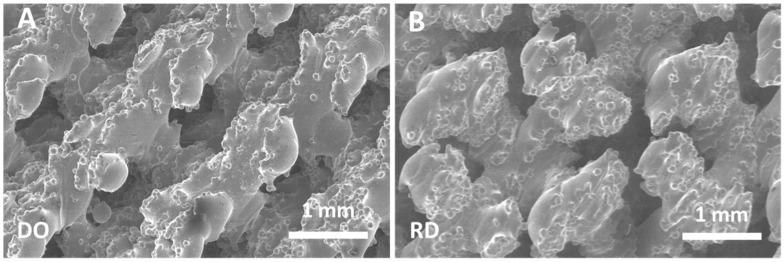
FESEM images of the scaffold’s top surface: (**A**) DO geometry, (**B**) RD geometry.

**Figure 5:**
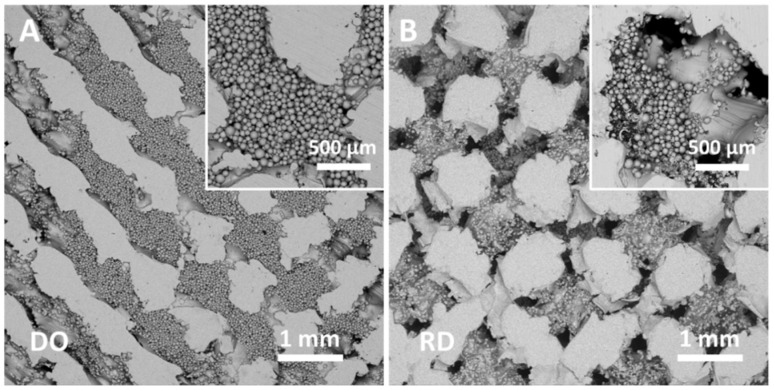
SEM images of the scaffold’s inner structure: (**A**) DO geometry, (**B**) RD geometry. Insets show the same area of sample at higher magnification.

The structure of the scaffold’s top surface as well as the scaffold’s inner structure were observed by FESEM and SEM, results are reported in Figure 4 and Figure 5. Morphology of the scaffold’s top surface is shown in Figure 4A for DO and Figure 4B for RD, while Figure 5A,B report the scaffold’s inner structure.

Independently of geometry (DO or RD), the top surface of the scaffold is formed of partially melted particles (Figure 4A,B) with well interconnected open pores.

The scaffold’s inner structure showed pores partially filled with unmelted and neck connected powder particles (Figure 5A,B).

Images in Figure 5 were taken in the central part (core) of the cut scaffold at the same magnification for comparison. High density residual powder in DO geometry (Figure 5A) with total occlusion of most pores was clearly evident. In contrast, RD geometry (Figure 5B) had pores only partially occluded. It is worth noting that SEM observations of the peripheric regions, far from the scaffold core, show open pores free from residual powder.

#### 3.2.2. Porosity and Residual Powder Volume

XCT measurements carried out on scaffold slices free from residual powder provided identical scaffold porosity values for all geometries, within experimental uncertainties. From porosity values, the total mass of struts (m_Ti_) and total volume of residual powder were obtained, according to Equation (1). The results are summarized in Table 5 for the scaffold geometries investigated.

From the data in Table 5, it was evident that the amount of residual powder inside the scaffold strongly depended on geometry, with DO containing about double the quantity of residual powder compared to RD. The relative density (*ρ* = 1 − *p*), where *p* is the scaffold porosity, as defined by Zhou et al. [42], could be also estimated from data in Table 5. For both geometries, the relative density *ρ* result was 37%.

#### 3.2.3. Structural and Chemical Characterization

Experimental chemical composition of the scaffolds obtained by EDS analysis was almost the same for both DO and RD geometries, the results are reported in Table 6. It is worth noting that the average chemical composition of the scaffold showed a slight decrease of vanadium with respect to the experimental composition of P1 and P2 powders (Table 4).

**Figure 6:**
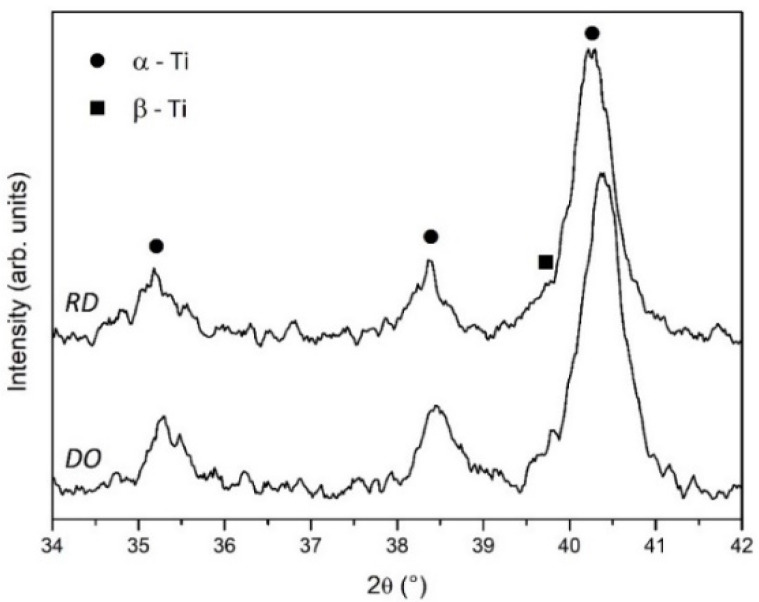
XRD patterns of DO and RD scaffolds. Full dot—α-Ti, full square—β-Ti.

Figure 6 shows XRD patterns of DO and RD scaffolds. The patterns reported the peaks of α-Ti (ICDD file n. 44-1294) and β-Ti (ICDD file n. 44-1288) phases as full dots and full squares, respectively. Scaffolds showed low intensity and broad diffraction peaks suggested low crystallization. Residual stresses and/or variations in crystalline state of scaffolds could be responsible for the slight angular peak shift visible in Figure 6.

#### 3.2.4. Mechanical Tests

**Figure 7:**
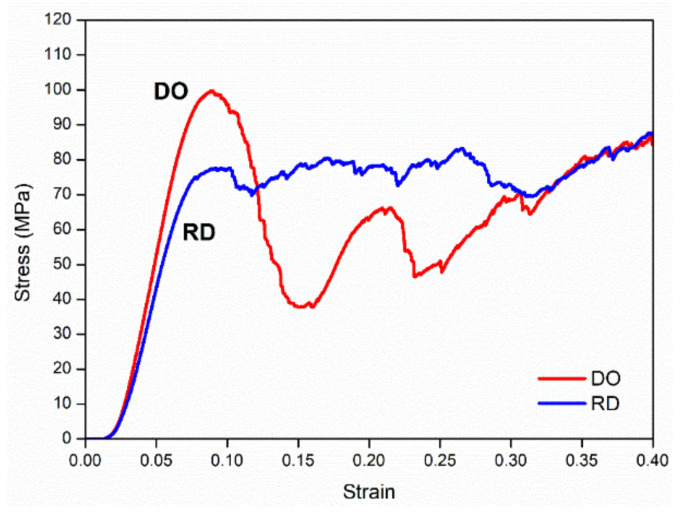
Mechanical behavior of scaffolds under compression for DO and RD geometries.

Compression tests carried out on scaffolds provided the mechanical behavior shown in Figure 7 for DO and RD geometries. The gradual collapse of scaffold elementary unit cells arranged on the scaffold layers was clearly evident in the DO curve, up to 40% reduction of initial scaffold height, which corresponded to a displacement of 4 mm.

In the RD curve the gradual collapse of scaffold elementary unit cells was less evident, although compaction occurred at about the same value of compression on initial height.

On compression, scaffolds showed a “barrel” phenomenon during layer compaction, with mechanically different behaviors along the x, y and z directions.

Maximum stress value at first peak was assumed as compression strength, after Li et al. [5].

Stress–strain curves in Figure 7 also allowed the calculation of Young modulus and compressive strength at 40%. Results are summarized in Table 7 with values obtained by Li et al. [5] and Ahmadi et al. [43] for comparison. Table 7 also shows reference values for cortical bone. It is worth underlining that the values of porosity and relative density reported in Table 7 for this work referred only to struts without any contribution from residual powder. Therefore, all values in Table 7 can be reliably compared.

#### 3.2.5. Surface Functionalization

**Figure 8:**
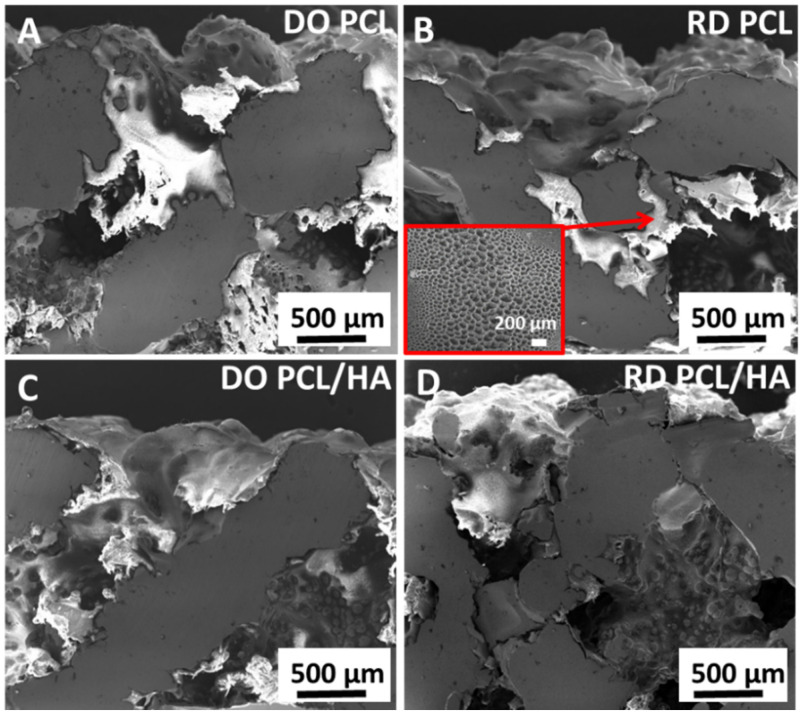
Micrographs of DO and RD scaffold coated with (**A**,**B**) PCL and (**C**,**D**) PCL/HA.

SEM observations in cross-section of PCL and PCL/HA coatings are shown in Figure 8. DO and RD scaffolds coated with PCL are reported in Figure 8A,B, respectively, while DO and RD scaffolds coated with PCL/HA are shown in Figure 8C,D for comparison. In SEM images the coatings appeared brighter than the metal struts of scaffold, continuous and well-adhered to the scaffold’s top surface as well as to surface of inner struts, when the coating penetrated inside the scaffold. Coating reduces scaffold surface roughness resulting in a smoothing effect leading to thickness variation of coating from a few tens of microns up to about 1 mm. The coating on the top surface always exhibits a porous structure due to solvent evaporation (inset in Figure 8C), as already reported by Abdal-hay et al. [44].

#### 3.2.6. Surface Roughness

**Figure 9:**
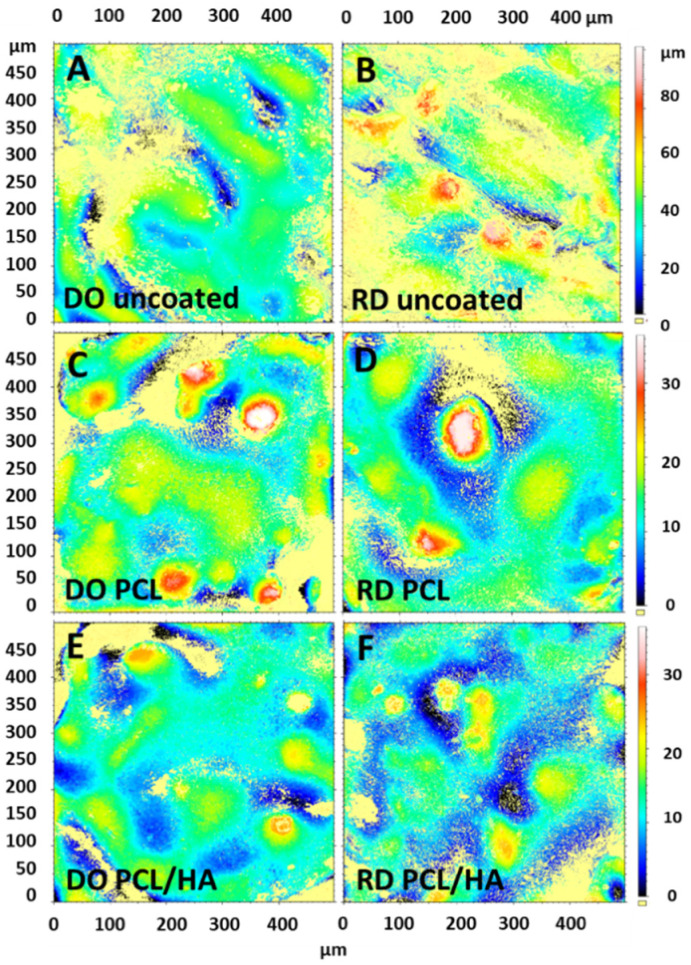
Roughness maps of DO and RD (**A**,**B**) uncoated, (**C**,**D**) coated with PCL and (**E**,**F**) with PCL/HA.

Top surface roughness maps of uncoated and coated scaffolds are reported in Figure 9 for comparison. From the roughness maps the roughness parameters of both scaffold geometries in uncoated and coated conditions were derived. The scaffold roughness parameters that estimated statistical distribution of height values along z axis, are listed in Table 8, with: Ra—average surface roughness, mean value of absolute distances in the roughness profile; Ssk—surface skewness, symmetry of roughness profile with respect to the mean line; Sku—surface kurtosis, sharpness of roughness profile; Sp—surface peak, maximum height of roughness profile from the mean line; Sv—surface valley, maximum depth of roughness profile from the mean line. Average surface roughness (Ra) shows the same trend for DO and RD, higher values for uncoated scaffolds and a slight decrease for PCL and PCL/HA coated scaffolds. All scaffolds, except for the DO uncoated condition that has valley predominance (Ssk < 0), show predominance of peaks in the roughness profile (Ssk > 0), with higher values of maximum peak height (Sp) and maximum valley depth (Sv) for the RD uncoated condition. Sharpness of roughness profile (Sku) is higher for PCL than for PCL/HA coating regardless of scaffold cell geometry.

### 3.3. Scaffold Interaction with hMSCs

In order to evaluate scaffold interaction with hMSCs, all groups were examined for cell viability, morphology and activation of the FAK protein typically involved in the cell adhesion process.

**Figure 10:**
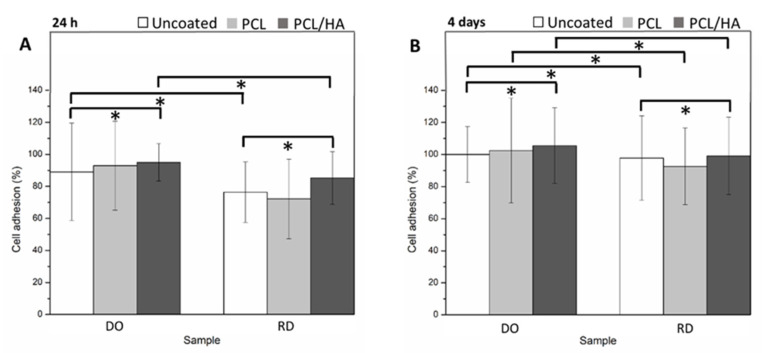
Cell viability at (**A**) 24 h and (**B**) 4 days on DO and RD uncoated, coated with PCL and with PCL/HA—*p* < 0.05 *.

Tests of cell viability in Figure 10 were carried out on DO and RD scaffolds in uncoated and coated condition, after 24 h (Figure 10A) and 4 days of incubation (Figure 10B). Data were normalized on the control (TCPS) and are reported as percentage values.

**Figure 11:**
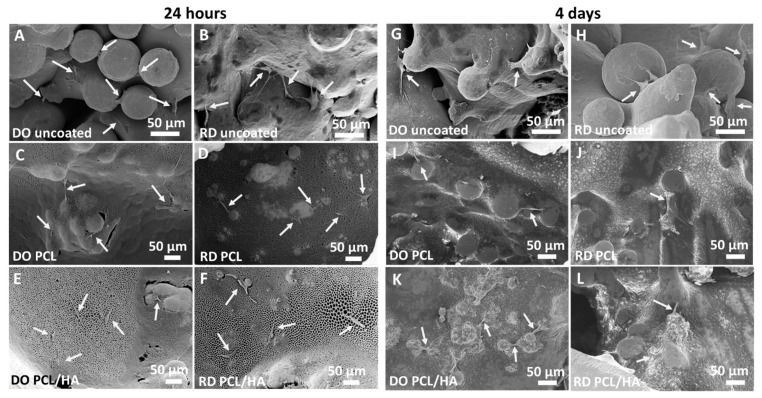
hMSCs after 24 h of incubation on DO and RD (**A**,B) uncoated, (**C**,**D**) coated with PCL and (**E**,**F**) with PCL/HA and hMSCs after 4 days of incubation on DO and RD (**G**,**H**)uncoated, (**I**,**J**) coated with PCL and with (**K**,**L**) PCL/HA. The hMSCs are indicated by the arrows.

The results of SEM observations performed in the same experimental setup on DO and RD scaffolds in uncoated and coated conditions after 24 h and 4 days of cell incubation are reported in Figure 11. After 24 h incubation, the cell distribution in the uncoated scaffolds are shown in Figure 11A,B for the DO and RD geometry, respectively. Images of the DO and RD scaffolds coated with PCL are shown in Figure 11C,D, respectively. Images of the DO and RD scaffolds coated with PCL/HA are shown in Figure 11E,F, respectively. In all scaffold conditions (coated and uncoated) and geometries (DO and RD), effective hMSCs presence was evidenced, while cell morphology suggested that after 24 h incubation, cells were in the spreading phase on the scaffold surface.

After 4 days incubation cell number had increased, with cells starting to colonize scaffold inner layers. Although, in DO and RD uncoated condition (Figure 11G,H) cells were clearly visible on the scaffold surface, they were not visible on the top surface of the coated scaffolds. In order to observe cell colonization of the scaffold inner part, coated scaffolds with PCL and PCL/HA were cut at 2 mm depth from the top surface and then observed by SEM. In the scaffold’s deeper layers, SEM observations clearly evidenced cells in spreading phase independently of scaffold geometry (DO or RD) and coating type (PCL or PCL/HA). The results of SEM observations of the coated scaffold inner region are reported in Figure 11I,J for DO and RD coated with PCL, and in Figure 11K,L for DO and RD coated with PCL/HA.

**Figure 12:**
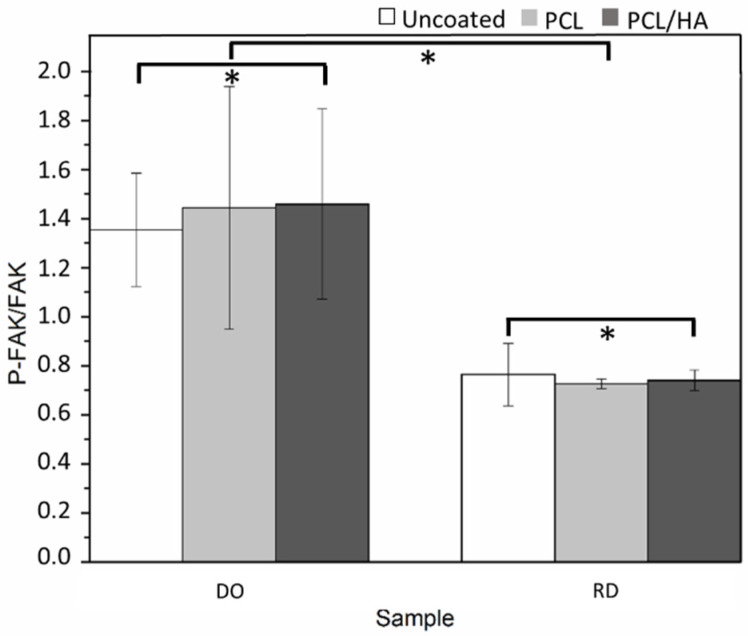
The activation level of FAK was measured by ELISA assay after 24h of incubation onto the different scaffolds. Bar graphs show P-FAK values normalized on the total FAK—*p* < 0.05 *.

ELISA assay analysis of FAK phosphorylation in hMSCs revealed that P-FAK was highly upregulated in cells grown on DO compared with cells grown on RD, indicating activation of FAK signaling by geometry cues (Figure 12).

## 4. Discussion

The ideal scaffold for tissue regeneration must: (a) support the normal cellular activity without producing any harmful effect (biocompatibility), (b) allow cells to adhere, proliferate and form extracellular matrix on its pore and surface, (c) allow formation of blood vessels to support the transport of oxygen, nutrients and waste products. Therefore, obtaining an ideal scaffold means taking control of any aspect of the production chain starting from the selection of raw materials and production technology to the design of pore shape, size and interconnectivity. Furthermore, cell viability tests allow the checking of material biocompatibility and cell colonization of the produced scaffolds. Therefore, in the perspective of tissue regeneration, biological characterization is as essential as mechanical and structural characterization.

In this study, cell viability tests were used as a fast check of biological response concerning scaffold pore topology and surface functionalization coating. For this reason, viability tests were stopped after only 4 days of culture, even before stem cell differentiation.

The raw material used to fabricate the scaffolds was Arcam Ti6Al4V powder blended in an unknown proportion with the same powder after several recycles. Blending recycled powder used in previous EBM processes with virgin new powder is common practice in the additive manufacturing industry to reduce costs and waste [17]. Although the chemical composition (Table 4) and lattice structure (Figure 3) of the raw powder (P1) were almost unaltered with respect to the virgin new powder (P2), in agreement with Alamos et al. [45], SEM images clearly showed particle aggregation through neck formation in P1 (Figure 2A).

Powder sintering occurring during the preheating and melting steps of the EBM process led to the formation of necks between particles (Figure 2A), that were responsible for powder aggregation. Although aggregation increased powder strength [32], aggregated particles remained entrapped in the pores of scaffold core (Figure 5) with consequences on scaffold topology-connected performance and mechanical properties.

The amount of residual powder inside the scaffold depended on geometry as evident in Figure 5 and Table 5. In particular, DO geometry, which had more intricate pore pathway for the release of unmelted powder entrapped inside the scaffold [6], contained about 76% more residual powder than RD (Table 5).

The most important geometrical features in DO and RD geometries such as pore size, strut size and porosity were investigated by Li et al. [46] using software analysis. All these parameters not only affect biological performance but also play a central role in mechanical properties [5].

Li et al. [5,46] investigated scaffold produced from virgin new powder with the same unit cell geometries (DO and RD) of our scaffolds that were produced with the blended P1 powder described above. Therefore, results obtained by Li et al. from software simulation [46] and mechanical tests [5] were taken here as reference and compared with our results in Table 7.

In particular, Li et al. [5] estimated a porosity value around 70% for both DO and RD unit cell geometry (Table 7). In our case, the porosity experimental value was 63% for both geometries (Table 7). Discrepancies between software calculated (Table 2) and experimental values were fully due to the EBM manufacturing technique. In particular, the EBM processing parameters reported in Table 3 allowed scaffold to be obtained with a strut size much larger (ranging from 800 to 1000 µm for both DO and RD geometries) than the nominal CAD values listed in Table 2. This reduced scaffold porosity to 63% against the expected 80% of CAD calculation (Table 2). Nevertheless, the struts were fully dense without pores or lack of fusion defects (Figure 5).

The EBM process occurs in vacuum, thus avoiding any oxygen contamination that could modify the chemical composition and physical properties of the powder, even after reuse [32]. However, the melting process in EBM can induce compositional variations in the final parts with respect to the metal powder. In our case, this event was confirmed by EDS analysis of scaffolds whose average chemical composition (Table 6) showed slight decrease of vanadium with respect to the alloy’s nominal composition (Table 1) and the experimental composition of the blended powder (Table 4).

XRD analysis of P1 and P2 powders (Figure 3) clearly showed the joint presence of α-Ti and β-Ti phases in both specimens. The peak intensity in the patterns evidenced better crystallization and higher β-Ti content in P1, due to the recycled part of blended raw powder. XRD patterns of scaffolds (Figure 6) confirmed the joint presence of α-Ti and β-Ti phases suggesting that the vanadium decrease measured by EDS was incapable of deeply changing the alloy’s microstructure. Nevertheless, comparing the XRD patterns of powders (Figure 3) and scaffolds (Figure 6) a markedly reduced crystallinity and possible residual stresses in scaffolds could be argued.

During the melting step of the EBM process, the electron beam provides enough energy to induce localized powder melting. However, in regions adjacent to the melted zone, the powder temperature does not reach the melting value so that large numbers of spherical titanium particles remain partially bonded to the walls of the melted pool, giving rise to the surface morphology reported in Figure 4. Studies on surface roughness of EBM implants identified Ra = 24.9 μm as the upper limit value for positive effects on cell proliferation and differentiation [47]. Roughness values of the scaffold surface reported in Table 8 are well below this limit, even in uncoated condition, confirming that scaffolds manufactured with the EBM parameters reported in Table 3 meet the needs of scaffold−host tissue interface for bone regeneration. Moreover, surface functionalization coating further decreases average surface roughness (Table 8). Recent studies demonstrated beneficial effects in several biomedical applications of as-fabricated rippled surfaces in EBM processed alloys [48,49].

Concerning additional topology-connected performances of scaffolds, it is worth noting that the DO geometry generally provides a more suitable structure for cell vitality than RD, both at 24 h and 4 days. Metabolic protein quantification confirms the better biological response of DO against RD. Quantification of P-FAK on total FAK (Figure 12), being the phosphorylation of FAK, the activator signal of the cell adhesion pathway, is indicative of a gentler environment for cell adhesion. Regardless of the percentage of viable cells present in scaffolds, a higher value of P-FAK/FAK of DO geometry corresponds to a greater number of viable cells attached to the scaffold. Therefore, since cell adhesion is a regulator of cellular proliferation, migration and differentiation [50], this result suggests a better biological response of the DO unit cell geometry against RD, in perfect agreement with the numerical analysis by Li et al. [6].

Further improvement in cell adhesion and proliferation was obtained by coating the scaffold surface with a single layer of PCL or PCL/HA. For both scaffold geometries (DO and RD) surface coating enhanced cell viability at 24 h and 4 days, especially in the presence of HA (Figure 10). The PCL coating, with or without HA, promoted enhancement of P-FAK activation independently of the scaffold geometry (DO or RD).

Coatings (Figure 8) adhered to the substrate in varying thickness according to the surface topography developed by partially melted powder particles. Independently of coating type (PCL or PCL/HA), a porous structure formed on the coating surface due to solvent evaporation (inset in Figure 8B). This porous structure promoted nutrient diffusion and osteointegration, thus supporting cell differentiation and proliferation [44]. The coating changed the morphology and chemical composition of the scaffold surface, thus improving further surface biofunctions [18]. Moreover, coating reduced the surface roughness, PCL/HA had the lowest Ra value (Table 8) and improved the biological behavior of stem cells (Figure 10B). SEM observations at 4 days incubation showed an absence of stem cells on the scaffold’s coated surface, suggesting penetration of hMSCs inside the scaffold (Figure 11). Therefore, the investigated coatings enhanced cell penetration into the scaffold, with PCL/HA showing better performance due to lower surface roughness.

In terms of mechanical behavior under uniaxial compressive testing (Figure 7) scaffold unit cell geometry seemed to be the most influencing parameter, although powder aggregation effects in the scaffold core must be considered. Stress–strain curves shown in Figure 7 for DO and RD unit cell geometry exhibited a general shape in agreement with the results in literature for the same cell topology [5,43]. The DO curve grew rapidly up to maximum stress value, corresponding to the compression strength, then large oscillations due to collapse of unit cells under compressive load followed. The collapse mechanism was clearly described by Ahmadi et al. [43] and Del Guercio et al. [51] In contrast, the RD curve after its first rise showed small oscillations around the same average stress value (Figure 7), in agreement with the results in literature [5,43]. However, contrary to the results in literature, DO and RD curves in Figure 7 reached the same stress value in the final part of the curve at high strain values. The two curves were completely superimposed above 35% strain, when the unit cells were all collapsed, and the scaffold structure arrived at compaction (Figure 7). Ahmadi et al. [43] reported the trend of compression strength and yield stress as a function of relative density for DO and RD geometries, showing that for relative density below 25% the two curves were superimposed, while for higher values RD always exhibited higher mechanical properties against DO. Ahmadi et al. [43] attributes the lower mechanical properties of DO to the unit cell geometry which is relatively simple and different struts provide only limited support to each other during compression. The relative density of our scaffolds, obtained by experimental calculation of porosity from XCT analysis, was 37% with compression strength of RD (78 MPa) lower than DO (99 MPa), as reported in Table 7. Generally, the mechanical behavior of our scaffolds strongly differed from the results of Li et al. [5] and Ahmadi et al. [43] for similar structures (Table 7). In particular, the large size of struts (ranging from 800 to 1000 µm) in our scaffolds determined the elastic behavior (Young modulus) and compression strength of DO and RD geometries (Table 7). Following Ahmadi et al. [43], the more complex unit cell geometry (RD) exhibited lower elastic properties (Table 7), while absolute values of Young modulus were always lower than the values from Li et al. [5] for similar cell geometries with lower strut size and lower relative density (Table 7). Therefore, higher strut size gives rise to stiffer structures with different behaviors for different unit cell geometries. In our case, both strut size and the absence of structural defects inside the struts (Figure 5) were responsible for higher compression strength of DO with respect to RD (Table 7).

On compression, in the simpler DO geometry failure of a single strut caused collapse of an entire elementary unit cell, then producing the large oscillations visible in the DO curve in Figure 7. As the collapse of elementary unit cells occurred from outside to inside during compression, the first part of the stress–strain curve (Figure 7) was not influenced by the residual powder stored in the scaffold core (Figure 5). However, when all external elementary unit cells had collapsed, elementary unit cells in the scaffold core that were partially filled with sintered residual powder, started to deform. On collapse of core elementary unit cells, the scaffold’s mechanical properties became dependent on sintered residual powder, which increased compressive stress and stress on struts in the longitudinal direction perpendicular to the compressive force. In this way, differences in mechanical properties linked to different elementary unit cell geometry vanished, overruled by the presence of residual powder, so stress–strain curves of DO and RD unit cells coincided. It is worth noting that the compression strength at 40% of our scaffolds is very close to the experimental value of cortical bone (Table 7), suggesting that the presence of residual sintered powder did not deteriorate the mechanical performance of the scaffolds.

## 5. Conclusions

In biomedical industries, the high cost of device preclinical studies generates a gap between investigation and device commercialization. The aim of this study is to fill the gap by checking the feasibility of cost saving processes based on EBM technology. Starting from a mixture of reused and virgin new powders, as commonly done in industrial practice, scaffolds with diamond (DO) and rhombic dodecahedron (RD) elementary unit cells were produced and surface functionalized from the perspective of tissue regeneration applications. Surface functionalization was carried out by coating the scaffold with either PCL or PCL/HA layers. The feasibility of our industrial approach was verified by comparing scaffold biomechanical performance with standard preclinical studies in literature.

The main results obtained can be summarized as follows:

- EBM technology is suitable for the production of porous structures with controlled topology and well-defined unit cell geometry showing appropriate biological performance and surface characteristics for in vivo perspectives. Low surface roughness typical of the EBM process promotes hMSC proliferation, while the partially sintered residual powder in the scaffold’s core is responsible for the porosity mismatch between experimental and CAD design values;
- The mechanical performance of the scaffold is mainly influenced by elementary unit cell geometry. The presence of residual powder becomes evident for compression values outside the range of in vivo use of the device and tends to cancel out the differences between geometries. In general, the compression behavior of the scaffolds is quite similar to natural bone tissue and follows trends reported in literature, although strut size and the absence of structural defects inside the struts are responsible for the higher compression strength of DO against RD;
- Short-term cell viability and metabolic protein quantification identifies DO as a better biological environment than RD, in agreement with literature-reported numerical simulations. After 4 days incubation, hMSCs start to penetrate inside the scaffold, favored by adherent, continuous and protective layers of PCL and PCL/HA.

From the above results it is evident that powder recycling represents a convenient industrial practice applicable to the manufacture of biomedical devices to heavily reduce preclinical costs without altering biomechanical performance.

## Figures and Tables

**Table 1:** Nominal chemical composition (wt.%) of the Arcam Ti6Al4V powder in virgin new condition (P2).

Al	V	C	Fe	O	N	H	Ti
6.0	4.0	0.03	0.1	0.1	0.01	<0.003	Bal.

**Table 2:** CAD values of porosity and strut size for DO and RD geometries.

Geometry	Porosity (%)	Strut Size (µm)
DO	80	420
RD	80	460

**Table 3:** Arcam A2X printing parameters.

Printing Parameters	Outer Contour	Inner Contour
Maximum beam current (mA)	3	3
Focus offset (mA)	0	0
Speed (mm/s)	450	470
Offset (mm)	0.13	
Number of perimeters	1	1

**Table 4:** Chemical composition (wt.%) of P1 and P2 powders obtained by EDS analysis. AV—average value, SD—standard deviation.

Element	P1 (wt.%)		P2 (wt.%)	
	**AV**	**SD**	**AV**	**SD**
Al	5.8	0.2	6.1	0.4
Ti	90.60	0.14	90.5	0.2
V	3.60	0.11	3.4	0.2

**Table 5:** Experimental values resulting from XCT analysis and calculated values of residual powder weight (m_pw_) and volume (V_pw_). m_s_—total scaffold mass; V_s_—total scaffold volume; m_Ti_—total mass of struts; V_Ti_—total volume of struts; m_pw_—total mass of residual powder; V_pw_—total volume of residual powder.

Geometry	Porosity (%)	m_s_ (g)	V_s_ (cm^3^)	m_Ti_ (g)	V_Ti_ (cm^3^)	m_pw_ (g)	V_pw_ (cm^3^)
DO	63	1.94	1	1.64	0.37	0.30	0.0996
RD	63	1.81	1	1.64	0.37	0.17	0.0564

**Table 6:** Chemical composition of scaffold obtained by EDS analysis. AV—average value, SD—standard deviation.

Element	Scaffold (wt. %)	
	**AV**	**SD**
Al	6.0	0.5
Ti	91.7	0.7
V	2.4	0.2

**Table 7:** Experimental results obtained from the compression tests compared to the results obtained by Li et al. [5] and Ahmadi et al. [43] with the same scaffold geometries, and reference values for cortical bone.

Reference	Porosity (%)		Relative Density (%)		Young Modulus (GPa)		Compression Strength (MPa)		Compression Strength at 40% (MPa)	
	DO	RD	DO	RD	DO	RD	DO	RD	DO	RD
This work	63	63	37	37	1.949 ± 0.001	1.622 ± 0.001	99	78	86	88
Li et al. [5]	70	70	30	30	2.59 ± 0.13	4.89 ± 0.05	88 ± 5	174 ± 4		
Ahmadi et al. [43]	80	72	20	28			45	80	30	70
Cortical bone					3.9–11.7				80	

**Table 8:** Roughness parameters estimated from roughness maps.

Parameters	DO			RD		
	Uncoated	PCL	PCL/HA	Uncoated	PCL	PCL/HA
Ra (µm)	6.5	3.9	2.8	10	3.5	3.27
Ssk	−0.8	0.7	0.13	0.4	1.14	0.5
Sku	4.3	5.4	4.1	4.0	7.13	3.6
Sp (µm)	2.9	3.8	2.1	9.1	3.26	2.7
Sv (µm)	5.8	2.18	1.7	9.8	1.5	1.6

## Data Availability

Data sharing not applicable.
